# The interplay of crack hopping, delamination and interface failure in drying nanoparticle films

**DOI:** 10.1038/srep32296

**Published:** 2016-08-25

**Authors:** Bin Yang, James S. Sharp, Mike I. Smith

**Affiliations:** 1 School of Physics and Astronomy, University of Nottingham, Nottingham, NG7 2RD UK

## Abstract

Films formed through the drying of nanoparticle suspensions release the build-up of strain through a variety of different mechanisms including shear banding, crack formation and delamination. Here we show that important connections exist between these different phenomena: delamination depends on the dynamics of crack hopping, which in turn is influenced by the presence of shear bands. We also show that delamination does not occur uniformly across the film. As cracks hop they locally initiate the delamination of the film which warps with a timescale much longer than that associated with the hopping of cracks. The motion of a small region of the delamination front, where the shear component of interfacial crack propagation is believed to be enhanced, results in the deposition of a complex zig-zag pattern on the supporting substrate.

The drying of colloidal dispersions has strong relevance to many industrial coating processes such as those involved in the deposition of paints, inks and specialty coatings^1^. During the drying process, a coating can fail in numerous ways as a result of the build-up of stresses in the films. The mechanisms by which these stresses are dissipated take different forms; including the formation of cracks, delamination of the coating from the substrate and the formation of shear band like structures within the films. The complexity of the drying process and the stress distributions that build up in these coatings have meant that providing an accurate theoretical description of the failure mechanisms in colloidal nanoparticle films has been challenging. However, a number of authors have studied how factors such as film thickness^2^,^3^, particle size^4^,^5^, drying rates^6^,^7^ and substrate constraints^8^,^9^ influence the failure mechanisms in thin nanoparticle films. Delamination is one such failure mechanism which can undermine the physical stability of a coating and occurs when a film debonds from its substrate. This type of failure mechanism can occur during the drying of a nanoparticle suspension and has been studied previously by a number of authors^7^,^10^,^11^,^12^,^13^.

As nanoparticle suspensions dry, a film is formed as the result of the evaporation of the suspending fluid. The details of the processes involved in the deposition of particles are subtle and complex, leading to a number of distinct regions in the drying film which are shown in Fig. 1(d) and are discussed in more detail below.

When the nanoparticle suspension is spread on a substrate, a film begins to form at the edges of the drying fluid. Evaporation of the suspending fluid then results in a quasi-steady state being set up in which distinct regions can be clearly observed behind the retreating front of liquid (far right Fig. 1). Immediately behind the front of liquid, a convective flow carries particles towards the film^14^, where they undergo solidification in a region called the compaction front^15^. This compacted region tracks the motion of the front of liquid and consists of a close packed film of particles with water in the interstitial spaces. This region of the film remains saturated as a result of the capillary pressure generated by small liquid menisci between particles at the surface which draw liquid through the film of close packed particles^5^. These capillary forces also place the entire film under stress and are responsible for driving many of the interesting failure mechanisms that are observed in drying films of colloidal nanoparticles e.g shear bands, cracks and delamination.

Immediately behind the compaction front, lines known as shear bands are often observed to form at an orientation of 45 degrees to the direction of drying^6^,^16^. These plastic deformations occur in a relatively narrow band in the film, within which particle rearrangements take place^6^,^16^. A little further behind the compaction front, the film is observed to form cracks that propagate parallel to the drying direction. These cracks form as a result of stresses that build up in the film due to substrate constraints, which confine the drying film and leave it in a state of tension^8^,^9^. Dufresne *et al.*^17^ observed similar cracks to the ones shown in Fig. 1 and showed that they do not follow the compaction front smoothly, but rather that they move somewhat erratically by hopping in discrete steps. This hopping behaviour was explained in terms of the difference in stress required to initiate and arrest a crack tip during the drying process.

Further from the compaction front, the film may delaminate from the surface and become curved. This delamination occurs when the strain energy stored in the film by drying and compaction of particles overcomes the adhesion energy holding the film in contact with the substrate^7^. These drying stresses also result in plastic deformations in the film during the delamination process and often result in a permanent deformation with a characteristic radius of curvature that depends on the film thickness and drying rate^18^. Sarkar *et al.* studied the delamination of silica suspensions drying in a capillary tube^11^. These authors showed that the delamination front moved in an intermittent manner, similar to that observed for crack propagation.

The interplay between the processes of drying, compaction, delamination, cracking and shear band formation result in the formation of some fascinating structures within drying colloidal nanoparticle films. What at first appears to be a relatively simple system i.e. particles suspended in water, gives rise to a complex range of phenomena which have thus far evaded a complete and thorough explanation. In this study we aim to show how the phenomena of shear bands, crack propagation and delamination described above are inter-connected and that their dynamics are strongly correlated.

## Results and Discussion

**Figure 1:**
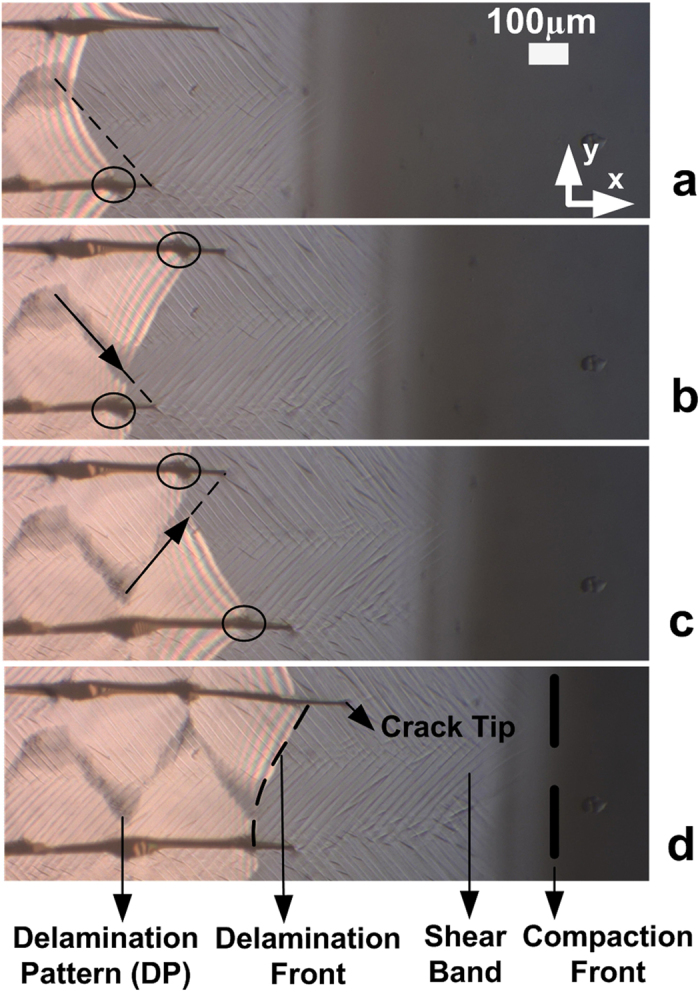
Time lapse images taken using an inverted microscope in reflection mode. The image sequence covers a period of 10 minutes. In image (**d**) the distinct regions of the drying process described in the text are labelled. The dark zig-zag pattern appears first at the delamination front (bright fringes) resulting from the adhesion of a thin layer of the film to the substrate. The cracks hop intermittently resulting in a breaking of the symmetry following the hopping of a crack. The delamination pattern can be seen to move towards the trailing crack tip, changing direction when this trailing crack hops further forward. Once the delamination front has moved to a new position it exerts a force on the crack, causing it to widen where the delamination front becomes pinned. These regions are circled in each image.

Figure 1 shows four time lapse inverted microscope images that were collected in reflection mode over a period of approximately 10 minutes, (See also supplementary movie 1). This figure shows that the crack tips (travelling horizontally from left to right) are not all the same distance from the compaction front. In fact, these crack tips were observed to hop in a discrete manner to new positions one after the other. As the supplementary movie shows, the crack tips are followed by the delamination front, which begins where the coloured interference fringes appear on the left hand side of each image. In all cases the delamination front followed the crack tips and each piece of delaminating film was separated from adjacent pieces by a crack; thus isolating it to some extent from its neighbours.

There are several intriguing features of the delamination process. Firstly, the delamination front was always curved and displayed asymmetric characteristics which tracked behind the motion of the crack tips. When a crack was observed to hop (Fig. 1(a)), a small delay occurred before the delamination front began to move (Fig. 1(b)). The delamination then occurred in such a way that the delaminating front was furthest forward on the side of the most advanced crack tip (Fig. 1(c,d)).

**Figure 2:**
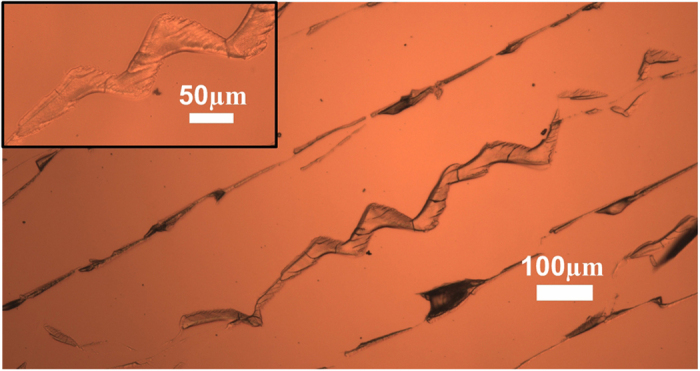
Deposits left behind on the substrate after delamination of a silica film. Two types of surface structure are observed. A pattern which matches the dark zig-zag line observed in the drying films, and deposits which match the location of the cracks in the film. The deposits near the positions of the cracks were not uniform in width. The wider sections arise from the features in Fig. 1 which are formed as the crack slowly widens under the influence of shear stresses in the film.

During delamination a dark shaded region was present at the apex of the curve produced by the delamination front. This dark region was observed to zig-zag in the delaminated section of the film (as shown in Fig. 1). From here on we refer to this feature as the delamination pattern or DP. This DP was not visible when viewed in reflection mode on an upright microscope because of the opaque nature of the films, but it *was* visible when viewed in reflection mode on an inverted microscope. This suggests that this feature was only associated with structures on the underside of the film. When the films were removed from the glass, patterns that match the DP were also observed on the substrate (see inset Fig. 2).

In a recent study by Giorgiutti-Dauphine *et al.* it was reported that a delaminating colloidal film sometimes leaves an array of evenly spaced fine lines behind due to a Saffman-Taylor like instability^12^. These authors reported that the line deposits were perpendicular to the delamination front and were spaced ~3–4 μm apart with a width of less than 1 μm. These values are consistent with the wavelength based on the predictions of a Saffman-Taylor instability for a delaminating colloidal film^12^. In contrast, the DP structures observed here only have one such feature between two neighbouring cracks (crack spacing ~400 μm) and the features are ~50–100 μm in width. In the Saffman-Taylor instability, a viscous finger occurs as the result of a less viscous fluid (air) penetrating into a more viscous liquid (water/colloidal film). However, the curvature of the delamination front is in the opposite direction to that expected for a Saffman-Taylor type process at the point of pattern deposition in the process described here. The path of the DP observed in Fig. 1 was also observed to zig-zag along the film/substrate interface. The twisting and turning of this pattern was shown to arise not from an instability, but from the motion of the crack tips. This can be seen in Fig. 1 where the motion of the DP was always towards the trailing crack tip. It seems therefore that the DP in the samples described here is the result of a different type of mechanism.

To understand the motion of this delamination pattern in more detail the inter-relationship between different features that occur in drying films are studied. It is argued that the final positions to which cracks hop is influenced by the presence of shear bands, and that this in part leads to the asymmetry of neighbouring crack tips. The hopping of crack tips then initiates delamination in the adjacent film. In addition, the alternating delamination of the film in the region between two hopping cracks results in a concave in plane curvature of the film. In the regions where the DP was observed to form it is believed the propagation of the interfacial crack is modified, increasing the relative contribution of shear to peeling when compared to other regions on the delamination front. The resulting increase in the shear contribution in the apex region pins the delamination front and deposits material on the substrate at the apex of the delamination front. The motion of this point of the delamination front in response to the alternating crack hops then gives rise to the observed zig-zag of the delamination pattern.

### The influence of shear bands on crack hopping

Dufresne *et al.*^17^ showed that crack tip growth tracks the motion of the compaction front in an intermittent manner. They argued that cracks hop in a discrete way because of the different stresses required to initiate (σ_go_) and arrest (σ_stop_) fracture. Whilst a critical stress for crack propagation may be estimated from the Griffith’s criterion, this can only explain the origin of the crack initiating stress and leaves a description of the origin of the arresting stress lacking^17^. Assuming the existence of these two critical stresses, Dufresne *et al.* were able to produce a model which predicted a regular and consistent pattern of hops. However, this differs from the real situation where there is an apparent randomness in the final positions of cracks, despite the average behaviour obeying the predicted pattern. It was proposed that the stochastic nature of propagation could be explained in terms of changes in the shape of crack tips or as a result of inhomogeneities in the films.

In all of the experiments reported here, cracks were observed to hop discretely from one position to another as observed by Dufresne *et al.*^17^. It was observed that the shape of the crack tips showed little or no difference, after the propagation of the crack tip. (see Fig. 1). This suggests that spatial variations in the material properties of the films may be a more appropriate explanation for why cracks stop at particular, apparently random positions. However, this raises the question as to what factors are responsible for the inhomogeneities in the films?

Strong candidates for these inhomogeneities are the shear bands that form behind the compaction front. These shear bands result in the formation of diagonal lines at an angle of 45° to the drying direction and occupy approximately 10% of the volume and free surface area of the film^6^. In the experiments described here it was observed that hopping cracks almost invariably finish their movement on a shear band. Analysing the final position of cracks following hops, it was found that 90% of them terminated at a shear band. Shear bands are known to form as a result of plastic rearrangements in the film that shear the original close packed particle structures formed during drying. These shear bands also influence the local strain in the colloidal films^16^. Such a variation in the local strain (or packing of particles) might be expected to modify the local structure in the films and hence influence crack propagation.

To test the generality of the idea that shear bands influence the final positions of crack tips, crack trajectories were also studied in films containing 47 nm diameter polystyrene (PS) colloids. We chose to use polymer particles because they display the same qualitative crack hopping characteristics as drying silica nanoparticles, but the size of the gaps between shear bands is much larger which makes the dynamics easier to study.

**Figure 3:**
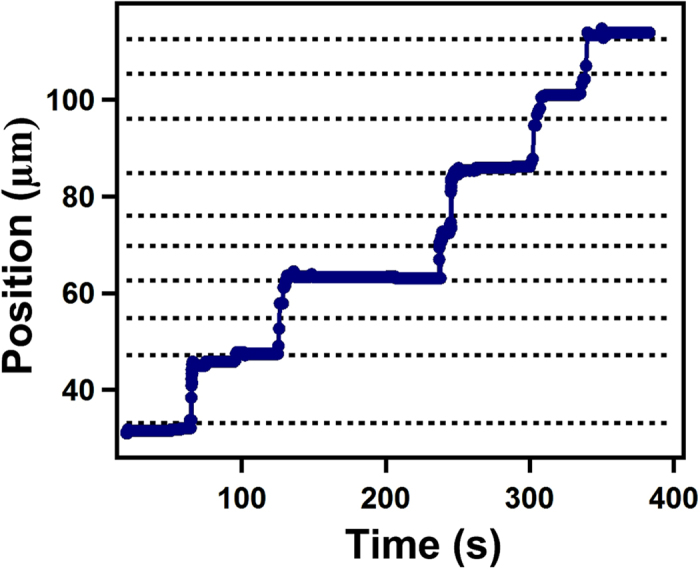
The influence of shearbands during crack hopping. The horizontal position of a crack tip (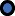) as it propagates through a drying film of 47 nm PS colloids with time. The position of all the shearbands present on the crack trajectory are shown as horizontal dashed lines. The crack tips are observed to hop between different positions that correlate well with the position of shear bands in the sample. Other inhomogeneities in the film may also stop the crack tip but the majority of crack hops finish at the location of shear bands.

Figure 3 shows how the horizontal position of a representative crack varies with time. The dotted lines show the horizontal position of shear bands that intersect the crack trajectory in the same film. While the cracks do not stop at every shear band, the influence that they have on the dynamics is clear. Despite occupying only a small volume of the film, the shear bands account for the majority of final crack positions, thus demonstrating their influence in the hopping process.

It is important to emphasise that other inhomogeneities in the film may also arrest the crack. Similar experiments performed with larger polymer particles and also for samples with added salt, where shear bands do not exist, also display sporadic crack arrest and hops. Since cracks do not stop at every shear band (see Fig. 3) it is also clear that shear bands only influence the motion of cracks in a material and are not a complete explanation of this phenomenon. A complete description of crack hopping is likely to depend on such factors as poroelasticity^19^ and a balance of the inertia and viscous dissipation that occur during crack propagation^20^. These would lead to increasing/decreasing stresses with time near the crack tip before/after hops^7^,^19^,^20^. However, shear bands seem to provide an explanation for the stochastic nature of the dynamics in small particles of both silica and polystyrene and in determining the final position of crack tips. The fact that crack tips stop in shear bands also indicates a significant variation in material properties near these locations in the film. Taken together these observations provide strong evidence that the stochastic nature of the crack hops is influenced by the presence of shear bands in a drying colloidal film.

### The role of crack hopping in the dynamics of delamination of the adjacent film

**Figure 4:**
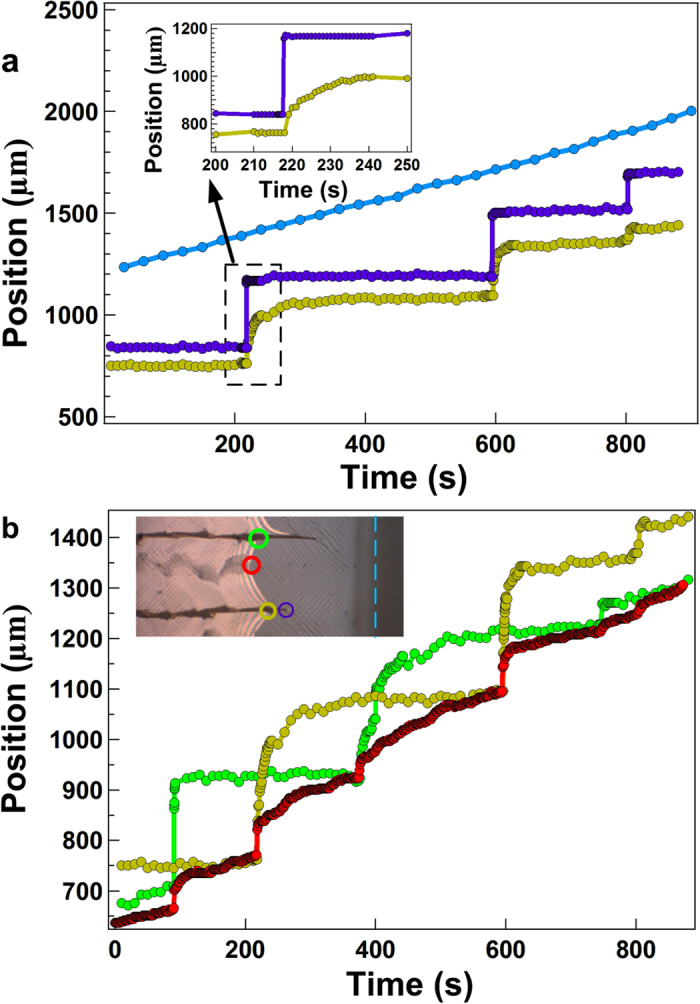
Relationship between the positions of the crack tips and the delamination front. Top and bottom panels show the same film but track the horizontal position of different features that are highlighted in the inset of the bottom panel. (**a**) The compaction front (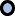) moves smoothly as the film dries. In contrast, a crack tip (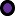) hops in discrete jumps. The film next to the crack tip (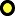) delaminates in response to crack propagation but over a longer timescale. The inset shows a close up of one particular crack hop and delamination event. (**b**) The delamination of both edges of the piece of film (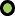,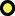) and the position of the delamination pattern at the point of delamination (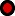).

Crack hopping is particularly important since it is this that controls the delamination dynamics of the adjacent film. Figure 4(a) shows a plot of the trajectory of the compaction front (light blue) and a single crack (purple) in a silica particle film, together with the position of the delamination front closest to the crack. This figure also shows that there is a hopping of the crack tips to new positions despite the fact that the compaction front moves slowly and continuously. It is clear from the synchronisation of crack hopping and delamination in Fig. 4(a) that the opening of the crack initiates the delamination process. This might be expected as models of delaminating films predict that the flaw size required to initiate delamination in a film reduces from ~20x the film thickness in the bulk, to around the film thickness of the material at a free edge^21^. However, the dynamics of the delamination of this edge are much slower than crack formation (see inset Fig. 4(a) and supplementary movie). Although there is some variability, the time scale for this delamination process is ~20 s which is significantly slower than the time taken for a crack to hop to a new position (<1 s, see inset Fig. 4(a)). This indicates that there is some additional relaxation process associated with delamination which may arise as a result of poroelastic phenomena^22^ or plastic deformations of the film^18^. Although delamination of the edge of the film occurs significantly more slowly than the hopping of a crack (see Fig. 3), additional changes to the film continue to occur over longer timescales ~400 s. For example, it was observed that the crack adjacent to the delamination front slowly widens, leaving a thin film of particles behind on the surface.

### Crack widening

Figure 2 shows micrographs of the film left behind after complete delamination of the film. It is likely that the thin cracks in the deposits occur after delamination of the film, as it dries out and are therefore not relevant to the following discussion.

For the image shown in the main panel, the motion of the drying front proceeded from right to left. Two types of structure can be observed in this image. Firstly, regions of film/particles left behind by the DP and secondly regions of film/particles left behind by the edges of each crack. Line profiles across these features show that in both cases they have thicknesses ranging from 50–140 nm.

The process by which pieces of film are left behind at each crack edge can be observed in Fig. 1. A number of points on each crack in Fig. 1 have been highlighted with rings. In Fig. 1b on the upper crack next to the position of the delamination front, the crack was observed to be much wider than the same crack observed a little earlier in Fig. 1(a). This indicates that the crack has been deformed in the intervening period. As the delamination front moved to a new position on the advancing crack it was observed that the crack at this new location widened over a period of ~100–200 s. This “crack widening” corresponds to the triangular sections deposited on the glass substrate (see Fig. 2). The crack widening begins when the delamination front has finished its forward movement and continues until the crack on the other side of the film hops forwards. During this time, there is a force acting along the delamination front which is perpendicular to the direction of delamination. The resulting force shears the film relative to the constrained layer in contact with the substrate. This process is slow and indicates a large resistance to motion. This observation is in agreement with predictions of theory and experiments on thin films which have shown that as delamination proceeds, the nature of the interfacial cracks shifts from one of peeling to a mode containing significant amounts of shear. Such a transition to a shear dominated mode results in a significant toughening of the interface which may arise from interfacial friction effects at the substrate or the need for plastic deformations to occur in the film for it to relax the applied stresses^21^,^23^,^24^,^25^.

### The Delamination Pattern

**Figure 5:**
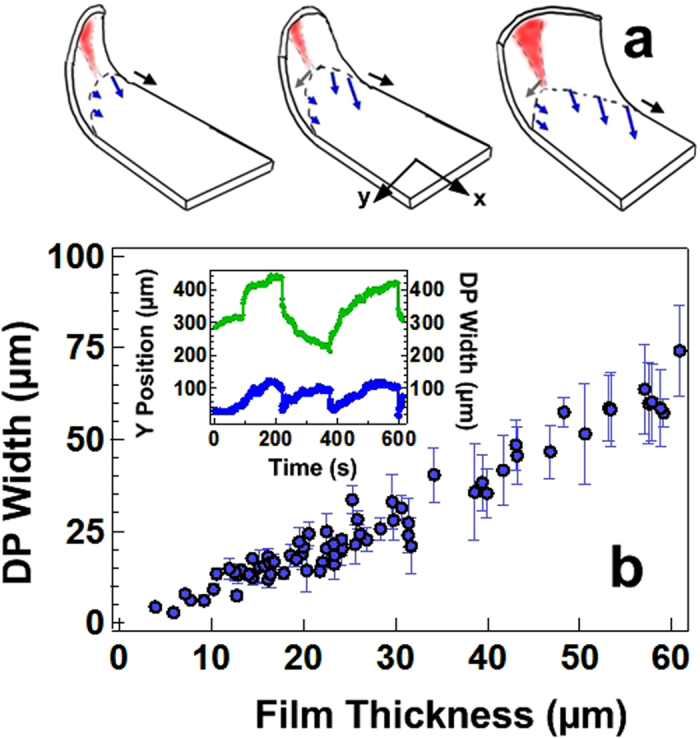
Deposition of the delamination pattern. (**a**) Schematic illustrating how the apex of the delamination front becomes compressed and moves in response to crack propagation and delamination of the adjacent film. As the apex moves towards the trailing crack tip more of the film can delaminate. The DP forms a zig-zag pattern as a result of the fact that it tracks the alternating motion associated with crack hopping in the film. (**b**) The width of the delamination pattern scales linearly with the film thickness. Inset) the y motion of the delamination pattern (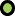) correlates well with changes in the width of the deposited pattern (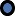).

The motion of the apex of the delamination front results in the deposition of a zig-zag pattern on the surface. This process, like the crack widening, is also slow compared to the initial delamination of the edge of the film. Figure 4(b) shows the same delamination data displayed in Fig. 4(a) (yellow circles) superimposed on the horizontal position of the delamination front in the region next to the neighbouring crack (green circles). The horizontal position of the DP at the point at which the film is delaminating is also plotted as the red circles. Figure 4(b) demonstrates that the motion of the DP is influenced by the delamination of each crack edge. This can be seen by the small sharp movement following each delamination event (Fig. 4(b)). However, the majority of the movement of the DP occurs slowly and over a longer timescale (~200 s) between these delaminating edge events, thus implying an enhanced resistance to delamination at the DP. Given the similarity of the film thicknesses left behind at the crack edge and by the DP, and the similar timescales associated with their formation, it seems a reasonable hypothesis that delamination in the DP region might also involve a shear contribution. Figure 5(a) shows a schematic of a delaminating film where one edge of the delamination front is moving forward following the hopping of a crack. Film shrinkage, induced by evaporation from the surface places the film under strain, resulting in a bending moment which drives the delamination^18^. During delamination one side of the film peels inwards, whilst the other edge remains relatively static (see Fig. 3b and supplementary movie). If one considers the direction of propagation of each side of the film it is also clear that a small region at the apex (shaded red in Fig. 5a) is placed under shear and compression during this asymmetric peeling. The DP is always located at the point where the apex between the two delaminating regions meet and occurs as the result of a complex mixture of shear, compression and peeling at this location.

In a detailed finite element study of the delamination front in “telephone cord” blisters, Faou *et al.*^25^ considered the interplay of adhesion and curved delamination fronts. Their calculations showed that at the apex of the curved region of a delaminating film, the mode of propagation of the crack becomes almost entirely shear (i.e. so called mode II). They were also able to demonstrate that the work of adhesion associated with debonding a film is strongly dependent upon the mode of crack propagation. Pure peeling results in a low work of adhesion which is more than compensated for by the elastic energy recovered during delamination. In contrast propagation via pure shear (as generated in the apex region) is not energetically favourable and results in the delamination front being pinned at this location.

This type of behaviour has also been observed experimentally during the delamination of polymeric strips, with a similar geometry to the one described in this work^26^. It was shown that the mixture of peeling and shear differed around the apex of the curved interfacial crack. This resulted in local pinning of the delamination front and a stick-slip type motion. The interfacial crack was also observed to deviate away from the interface leading to debris being left behind on the substrate.

These studies report many of the same phenomena observed in the experiments described here. Near the curved apex, the component of the stresses arising due to shear is expected to increase (ie mode II). During the crack widening process it was observed that shear resulted in the deposition of a thin film and it is reasonable to assume that shear could produce the same effect in the apex region of the delaminating films. Away from the apex, delamination has a stronger peeling component and the crossover between these two behaviours (shear and peeling) occurs when the crack mode dependent work of adhesion (G_c_) exceeds the energy gained from the elastic energy recovered from the film (G_0_)^25^. It appears that the cause of the delamination pattern appearing only at the apex region is the change in the mode of crack propagation at this location. This is supported by numerous theoretical studies that find that cracks kink away from the interface when the mode of crack propagation becomes dominated by shear^27^,^28^. A G_c_ larger than G_0_ near the apex would also explain why the delamination front becomes pinned at the apex, as it is energetically unfavourable to propagate the interfacial crack.

Figure 5b shows a plot of the width of the delamination pattern, measured for a wide variety of film thicknesses. The width scales linearly with film thickness. A full understanding of the delamination pattern requires a detailed knowledge of the stresses and strains near to the apex - which is particularly challenging given the asymmetry inherent in the delamination process and is beyond the scope of this study. However, a simple argument can be offered to show that that the observed scaling is commensurate with the picture outlined above.

As discussed above, the apex of the delamination front is located where the two sides of the delamination front meet. Where this occurs, the film is placed under a mixture of compression and shear and exhibits excess curvature (see Fig. 5a). The excess curvature at this point on the inner face of the delamination front leads to the backside of the film being placed in tension. When the tensile stress on the backside of the film exceeds the yield stress, this will cause the film to fail. This occurs via a mechanism that is somewhat similar to the buckling induced spalling considered by Evans *et al.*^29^ in which a bending film undergoes a transition to a higher curvature and may crack, or spall, on the outer face. The length scale of the folded region (*d*) for a delaminating film of thickness, *h*, can be related to the Young’s modulus, *E,* and the compressive stress, *σ*_*0*_, by the equation^29^.


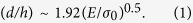


An upper bound for the stress, σ_0_, can be obtained from the capillary stress such that σ_0_  ~ 5.3 γ/a = 34 MPa^8^. Xu *et al.* have also measured the Young’s Modulus of films of the same type of silica particles that were used in this study and found it to be ~100 MPa^19^. These values predict d ~ 3.3 h, as opposed to the data in Fig. 5(b) where d ~ h. However, considerable uncertainty exists in determining the correct magnitude to use for the stress. Moreover, the prefactor in eq. 1 is dependent upon the exact geometry. In addition, the length scale, *d*, relates to the size of the folded region of the film not the cracked region which would give rise to the DP. However it is reasonable to assume that the size of the spalled section of the film that would result in the formation of the DP scales with the length of the folded region. Such an assumption provides an estimate of the scaling of the width of the delamination pattern with film thickness and predicts a linear scaling between the width of the DP and *h* according to Eq. 1. Figure 5b shows that measurements of the width of the DP do indeed scale linearly with film thickness. However, it is worth emphasising that there are important differences between the model used in deriving Equation 1 and the experimental geometry considered here. A key difference being that the model discussed above neglects the role of adhesion - a factor that is considered in more detail by Faou *et al.*^25^. A more detailed knowledge of the adhesive and cohesive interactions experienced by the film, as well as details of the curvature that occur near the apex would be required before a more accurate prediction of the width of the DP could be obtained.

The fact that the delamination pattern is deposited only at the apex of the delamination front means that motion of the apex is controlled by the asymmetry of crack hops. When a crack hops, the DP is usually situated relatively close to the crack. The film that is adjacent to the crack then peels relatively quickly but only as far across as the delamination pattern. At this point, the motion of the delamination front becomes pinned and peeling forces then act on the region near the DP resulting in the lateral motion of the pattern towards the trailing crack tip. As the DP moves laterally, a larger area of the film is then allowed to delaminate, releasing stored strain energy in the film. As the cracks hop, alternate peeling from each corner causes the DP to track this motion and results in the observed zig zag pattern.

Figure 5(b) inset shows that the width of the delamination pattern (measured at the delamination front) is also correlated with the lateral motion by the DP across the delamination front. As the DP changes direction there is an increase in the width of the deposit. It is not clear whether this relates to a further increase in the shear component^28^ or whether it relates to the rising and falling stress accompanying each crack hop.

In conclusion, our experiments demonstrate the important links between shear banding, crack propagation and delamination in drying colloidal films. Shear bands play a role in influencing the positions to which cracks hop. The alternating nature of crack propagation results in delamination of each edge of the film in response to each crack hop with a timescale that is controlled by plastic deformations within the film. Over longer timescales widening of the crack and a slowing of the motion of the apex of the delamination front are observed. This results in the deposition of a thin film of material on the substrate which is believed to be the result of an increased shear component in compressed regions of the film near the apex

## Methods

Ludox silica suspensions (AS-40, diameter ~22 nm, 40 wt% solids content, Sigma Aldrich) with concentrations in the range 2.5–10 wt% were prepared by diluting the concentrated suspension using deionised water. These suspensions were deposited on glass coverslips to produce films with thickness values in the 20–60 μm range. Experiments were also conducted on 2.5 wt% solutions of PS colloids of diameter 47 nm. The suspensions were then spread on pieces of glass with the edge of a clean glass slide, resulting in a planar drying front. Prior to deposition all the pieces of glass were cleaned with detergent, ethanol, acetone and deionised water before being dried with nitrogen.

Images of the drying films were obtained using a Bruker inverted microscope equipped with a colour camera (Point Grey, Flea 3). All image analysis was performed using software written in Matlab. The thickness of deposited films was measured using a Dektak^3^ profilometer (Veeco instruments).

To measure the width of the delamination pattern, films of different thicknesses were dried on glass coverslips. The films which delaminated from the substrate, were easily removed with a gentle shake, leaving the delamination pattern and film near the crack edges behind on the surface. Microscope images were taken using a 10x objective. At particular locations the width of the delamination pattern was measured 5x to capture the variations, together with the crack spacing. The measured relationship between crack spacing and film thickness (λ ~ 5.5 h) was then used to estimate the film thickness.

## Additional Information

**How to cite this article**: Yang, B. *et al.* The interplay of crack hopping, delamination and interface failure in drying nanoparticle films. *Sci. Rep.* **6**, 32296; doi: 10.1038/srep32296 (2016).

## Supplementary Material

Supplementary Information

Supplementary Movie 1
