# Supplementary material for: The interplay of crack hopping, delamination and interface failure in drying nanoparticle films
Source: Sci Rep. 2016 Aug 25;6:32296. doi: 10.1038/srep32296 (PMC4997629; doi:10.1038/srep32296)
Supplement: Supplementary Information [file srep32296-s1.pdf]

Supplementary Information

The interplay of crack hopping, delamination and interface failure in drying nanoparticle films.

Bin Yang, James S. Sharp, Mike I. Smith*

School of Physics and Astronomy, University of Nottingham, Nottingham, UK, NG7 2RD

Corresponding author: mike.i.smith@nottingham.ac.uk

Supplementary Movie 1

Movie of the delamination process, showing how crack hops influence delamination which in turn leads to the deposition of a pattern at the apex of the delamination front.
